# Correlations Between *H. pylori* Gastric Histopathology and NAFLD: A Retrospective Observational Study

**DOI:** 10.3390/life15081309

**Published:** 2025-08-18

**Authors:** Ioana Alexandra Cardos, Cătălina Dănilă, Ovidiu Laurean Pop, Andrea Pop-Crisan, Ovidiu Pavel Burta, Andreea Camarasan, Felicia Marc, Simona Daniela Cavalu

**Affiliations:** Faculty of Medicine and Pharmacy, University of Oradea, 410073 Oradea, Romania; cardos.ioanaalexandra@student.uoradea.ro (I.A.C.); drovipop@gmail.com (O.L.P.); daniela.cavalu@didactic.uoradea.ro (S.D.C.)

**Keywords:** *H. pylori*, liver fibrosis score, atrophic gastritis, histopathology, NAFLD

## Abstract

The importance of *H. pylori* infection in the development of non-alcoholic fatty liver disease, liver fibrosis, cirrhosis, insulin resistance, and non-alcoholic steatohepatitis has been shown in earlier studies. Our work aims to assess the risk of developing hepatic fibrosis in patients with or without *H. pylori*, using noninvasive scores such as the APRI index, the BARD score, or the FIB-4 index, and to evaluate a possible association between the severity of fibrosis scores and histopathology evidence (such as chronic gastritis, gastric atrophy, gastric metaplasia, and gastric dysplasia). Moreover, the risk of preneoplastic stomach lesions was assessed in patients with hepatic fibrosis. The study enrolled a total of 110 patients: 65 were *H. pylori*-positive and 45 were negative. The differences in BARD, APRI, and FIB-4 indexes between *H. pylori*-positive and negative cases were assessed using the Mann–Whitney test. Noticeably higher BARD scores and APRI indexes were observed when comparing *H. pylori*-positive patients with NAFLD to *H. pylori*-negative ones. In terms of the FIB-4 index, an insignificant increase was observed in *H. pylori*-positive versus *H. pylori*-negative patients. Multiple linear regression was performed for the BARD scores and APRI indexes, revealing further significant associations with age and *H. pylori* status. A substantial correlation was demonstrated between *H. pylori* and elevated hepatic fibrosis scores in individuals with NAFLD and gastritis, suggested by the complexity features of infection and the intricacies of histology.

## 1. Introduction

*Helicobacter pylori* is one of the most common causes of gastric pathologies. It was discovered by Barry Marshall and Robert Warren in gastric biopsies of patients suffering from chronic gastritis and ulcer disease [1]. They were awarded the Nobel Prize for their discovery in 2005 [2]. Since 1994, *H. pylori* infection has been categorized as a type 1 carcinogen by the International Agency for Research on Cancer. Numerous epidemiological studies conducted since then have verified the role of *H. pylori* in the development of stomach cancer [3]. In 90% of MALT lymphoma (marginal zone B cell lymphoma) cases, the cause is *H. pylori* infection. It was also shown that patients with this condition experienced regression, while an effective eradication therapy reduced the incidence of MALT lymphomas [4]. Since its discovery, *H. pylori* has been linked to illnesses in the hematologic system (iron deficiency anemia (IDA) [5], immune thrombocytopenia (ITP) [6], vitamin B12 deficiency, and low-grade gastric MALT), cardiac (coronary artery disease), metabolic (metabolic syndrome and insulin resistance, altered lipid profile), neurologic (ischemic stroke, Alzheimer’s disease, Parkinson disease, migraine headaches), and dermatologic systems (chronic spontaneous urticaria, rosacea) in addition to gastrointestinal issues [7,8].

There is evidence from recent research that *H. pylori* plays a role in the pathophysiology of certain liver illnesses [8,9]. The importance of *H. pylori* infection in the development of non-alcoholic fatty liver disease, liver fibrosis, cirrhosis, insulin resistance, and non-alcoholic steatohepatitis has been shown by numerous studies.

During an *H. pylori* infection, liver inflammation due to other causes might also worsen. Some authors consider that autoimmune disorders of the liver and biliary tract are generated by the *H. pylori* infection [9]. NAFLD, or non-alcoholic fatty liver disease, is a very common disorder that affects 25–30% of adults in Western countries. Liver fibrosis, cirrhosis, and non-alcoholic steatohepatitis (NASH) are among the consequences of non-alcoholic steatohepatitis (NAFLD), and they all have an impact on the incidence of morbidity and mortality [10]. According to a different study, *H. pylori* infection may be one of the factors that contributes to the pathogenesis of non-alcoholic fatty liver disease (NAFLD), and treating this condition may benefit from eliminating *H. pylori* [11].

The pathogenic mechanism behind this disease is still unknown. Not enough research has been carried out on the relationship between liver damage and the gut microbiota, which includes *H. pylori*. Some *Helicobacter* species can damage the liver by producing certain toxins [12]. Furthermore, *Helicobacter* invasion of the mucosa of the small intestine may lead to an increase in gut permeability and make it easier for bacterial endotoxins to reach the liver through the portal vein [13].

Usually, liver fibrosis is assessed by both invasive (liver biopsy) and noninvasive tests (NITs). While liver biopsy remains the gold standard for diagnosing and classifying liver fibrosis, its application is restricted due to invasiveness, procedural risks, patient readiness, sampling errors, and variability between different observers [14]. The number of noninvasive tests (NITs) used to evaluate liver fibrosis has increased exponentially during the last ten years. Hepatic fibrosis can be noninvasively assessed using ultrasound-based imaging (Transient Elastography (TE), FibroScan, Acoustic Radiation Force Impulse (ARFI) elastography), or serologic tests (AST to Platelet Ratio Index (APRI), Fibrosis-4 (FIB-4), NAFLD Fibrosis Score (NFS), FibroTest, Enhanced Liver Fibrosis (ELF) test, and enhanced Liver Fibrosis (ELF) test) [15]. Hepatic fibrosis in individuals with non-alcoholic fatty liver disease is frequently predicted by employing noninvasive techniques based on hepatic fibrosis scores, such as the FIB-4 index, the APRI index, and the BARD score [16].

One of the most efficient methods to predict liver fibrosis in NAFLD patients is the BARD score, considered to be advantageous, as it is a noninvasive tool. Three factors make up the BARD score: BMI ≥ 28 (1 point), AST/ALT ratio ≥ 0.8 (2 points), and the existence of diabetes (1 point). Hence, a score between 0 and 4 points is achievable [16,17,18]. These variables were merged in a weighted sum based on the results of forced entry logistic regression analysis to create the BARD score, a simple composite score for predicting advanced fibrosis. According to a study developed by Harrison et al., advanced fibrosis has a strong (96%) negative predictive value when BARD scores are equivalent to 0 or 1 [17], while a score of ≥2 was associated with advanced liver fibrosis [17,18,19].

The APRI index (AST to platelet ratio index) was suggested for use in the diagnosis of severe cirrhosis and fibrosis in viral chronic C hepatitis cirrhosis and NAFLD. APRI can be a helpful noninvasive substitute, as major fibrosis and cirrhosis are ruled out when the score is at least 1.5 or higher. APRI levels in NAFLD patients typically rise proportionally to the severity of fibrosis, according to a study conducted by Loaeza-del-Castillo et al. [20].

The original purpose of the FIB-4 index was to evaluate fibrosis in patients infected with HIV and HCV. The FIB-4 index can be calculated using age and three parameters from standard laboratory tests: platelet count, aspartate aminotransferase (AST), and alanine aminotransferase (ALT). This makes it appropriate for individuals with non-alcoholic fatty liver disease [21,22].

A FIB-4 index < 1.3 is categorized as low risk, while a FIB-4 index ≥ 2.67 is categorized as high risk of fibrosis [3,9]. The recommendation for referral from primary care clinicians to hepatologists is a FIB-4 index ≥ 1.3 [23,24].

The present study aims to assess the risk of developing hepatic fibrosis in patients with or without *H. pylori*, using noninvasive scores such as APRI, BARD, or FIB-4. Secondary, the aim of the study was to evaluate a possible association between the severity of hepatic fibrosis scores and the histopathological features of the gastric lesions, especially those with preneoplastic risk (such as gastric atrophy and metaplasia).

## 2. Materials and Methods

### 2.1. Patient Selection Criteria

The study protocol was authorized by the institutional board of the County Emergency Clinical Hospital Oradea, Bihor (Romania) (permission number 14687/28.04.2023) and conducted in accordance with the ethical principles of the Helsinki Declaration. This retrospective study was conducted over a period of 1 year, between December 2023 and December 2024, involving 110 patients registered in the Digestive Endoscopy Department.

Patients included in the study met the following criteria: age over 18, presenting dyspepsia (for example, nausea, heartburn, or epigastric pain), who underwent upper gastrointestinal endoscopy with biopsies, and were previously diagnosed with hepatic steatosis by ultrasound examination. Patients were consecutively selected from the endoscopy registry.

Exclusion criteria: patients with chronic viral hepatitis B, C, autoimmune hepatitis, drug-induced hepatitis and alcoholic hepatitis); patients with heavy alcohol drinking habits (more than 60 g/day for men and 20 g/day for women) for more than ten years; patients previously treated for *H. pylori* infection or receiving antibiotics, bismuth medicines, H2 antagonists, or proton pump inhibitors four weeks before were also excluded.

**Figure 1:**
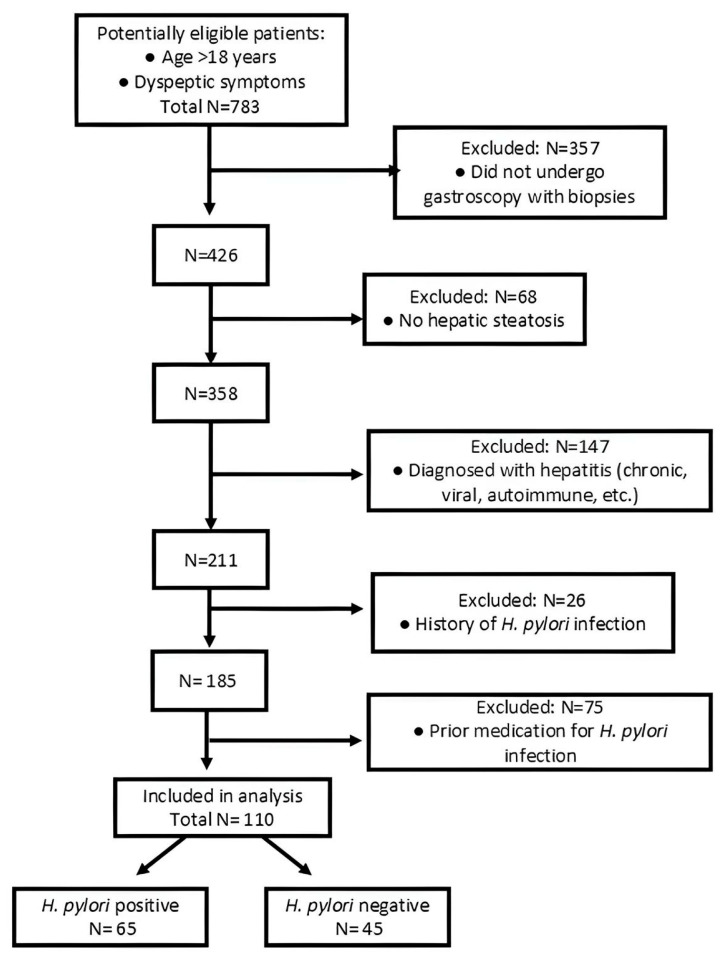
Flow chart of the selection criteria.

Based on the above criteria, a total of 110 patients were included, out of which 65 were *H. pylori*-positive and 45 were negative (Figure 1).

A physician clinically evaluated each patient, performed a body mass index (BMI), took a history of alcohol consumption, check for diabetes, and requested standard blood tests such as coagulation profile, complete blood count, liver function tests alanine aminotransferase (ALT), aspartate aminotransferase (AST), renal function tests, fasting blood glucose (FBG), low density cholesterol (LDL), high density cholesterol (HDL), triglycerides (TG), total cholesterol and serology for hepatitis B and C. Subsequently, noninvasive liver fibrosis scores were calculated: FIB4, BARD and APRI indexes.

All patients were examined by ultrasound using LOGIQ P9 ultrasound (GE Ultrasound Korea, Ltd., Seongnam, Republic of Korea, 2016) in order to diagnose NAFLD. Hepatic steatosis can be subjectively graded as absent, mild, moderate, or severe using B-mode ultrasonography. The commonly used grading scheme compares the liver’s echogenicity to the renal cortex, measures posterior beam attenuation, and assesses the visibility of the diaphragm and hepatic artery walls [25,26].

### 2.2. Endoscopy

One single endoscopist performed all the endoscopies using an Olympus Exera II CV 165 endoscope, Hamburg, Germany. The endoscopic results of intestinal metaplasia, gastric polyps, gastro-duodenal ulcer disease, atrophic gastritis, chronic and acute gastritis, and gastric neoplasia were all taken into account.

### 2.3. H. pylori Diagnosis Using the Rapid Urease Test

The *H. pylori* diagnosis was performed using the RUT test (AMA Co., Ltd., Lehmuskatu, Finland), which serves as the first-line indication for *H. pylori*, with 90% sensitivity and 95–100% specificity.

### 2.4. Histopathology

The histopathology analysis was performed by two skilled pathologists from the Pathology Department of the County Emergency Clinical Hospital, Oradea. The biopsies were mainly from the gastric antrum and evaluated in terms of glandular atrophy, metaplasia, reparative atypia, dysplasia, inflammatory mononuclear cellular infiltrates, and inflammation activity (neutrophilic infiltrations) [27].

Two sets of tissue sections were prepared for histological examination in order to identify *H. pylori* in the stomach mucosa. One set was stained with hematoxylin-eosin (H&E), while the other set was stained with Giemsa stain (Epredia-USA; Portsmouth, NH, USA) [28]. Four levels of inflammation—absent inflammation (Grade 0), mild inflammation (Grade 1), moderate inflammation (Grade 2), and severe inflammation (Grade 3)—were assessed using the Houston-updated Sydney system [29].

### 2.5. Blood Tests

All blood tests (ALAT, ASAT, platelets, lipid profile, glucose level, serology for hepatitis B, C) were performed in the same laboratory, utilizing the Abbott Alinity™ s System equipment (Abbott GmbH, Wiesbaden, Germany). The cut-off values used were as follows: 45 U/L for ALAT, 34 U/L for ASAT, 150.000–450.000/µL. for platelets, 82–115 mg/dL fasting blood glucose (FBG), 10–100 mg/dL for low density cholesterol (LDL), 40–60 mg/dL for high density cholesterol (HDL), 0–150 mg for triglycerides (TG), 0–200 mg/dL for total cholesterol and serology for hepatitis B and C.

The assessment of noninvasive liver fibrosis scores (FIB-4 index, BARD score, and APRI index) was conducted by considering the results of blood tests, platelet counts, the presence of diabetes mellitus, and the body mass index.

The following criteria were taken into account for the BARD score: BMI ≥ 28 (1 point), AST/ALT ratio ≥ 0.8 (2 points), and the existence of diabetes (1 point). Thus, a score between 0 and 4 points can be obtained. A score of 2–4 points indicates significant fibrosis. A FIB-4 index < 1.3 is categorized as low risk of fibrosis, while a FIB-4 index ≥ 2.67 is categorized as high risk of fibrosis. An APRI value of 0.3 and 0.5 rule out significant fibrosis and cirrhosis, while a value of 1.5 indicates significant fibrosis.

### 2.6. Statistical Analysis

The univariate analysis was employed in order to compare the differences in the BARD score, the APRI, and FIB-4 indexes between *H. pylori*-positive and negative cases, using the Mann–Whitney test. A Shapiro–Wilk test was performed prior to the application of the Mann–Whitney U test, confirming that the data did not follow a normal distribution (*p* < 0.05). The score differences identified as statistically significant were included in a multiple linear regression analysis, taking into account the variables such as age, gender, *H. pylori* status, and environment. Parameter estimates, standard errors, 95% confidence intervals, and *p*-values were calculated for each regression. Multicollinearity was assessed by calculating the Variance Inflation Factor (VIF) for each independent variable, and all VIF values were below 2, suggesting no significant multicollinearity between predictors. Additionally, the residuals from the regression models were visually inspected using Q–Q plots and residual vs. fitted plots to confirm homoscedasticity and approximate normal distribution of residuals. Correlation analysis was performed for continuous variables in our dataset, and Spearman coefficients were calculated. GraphPad Prism 9.3.0 was used for statistical analysis and figure generation.

## 3. Results

### 3.1. Baseline Characteristics

Our first goal was to evaluate the prevalence of *H. pylori* in the examined patients. We noticed that the infection was present in 59,8% of the patients, mostly men.

The mean age was 62 years for *Helicobacter*-positive and 63 years for *Helicobacter*-negative, most of them coming from urban areas. We calculated *p*-values using the Chi-square test for categoric variables, respectively, the Mann–Whitney test for continuous variables (Table 1).

### 3.2. Association Between Liver Fibrosis Scores and H. pylori Infection

**Figure 2:**
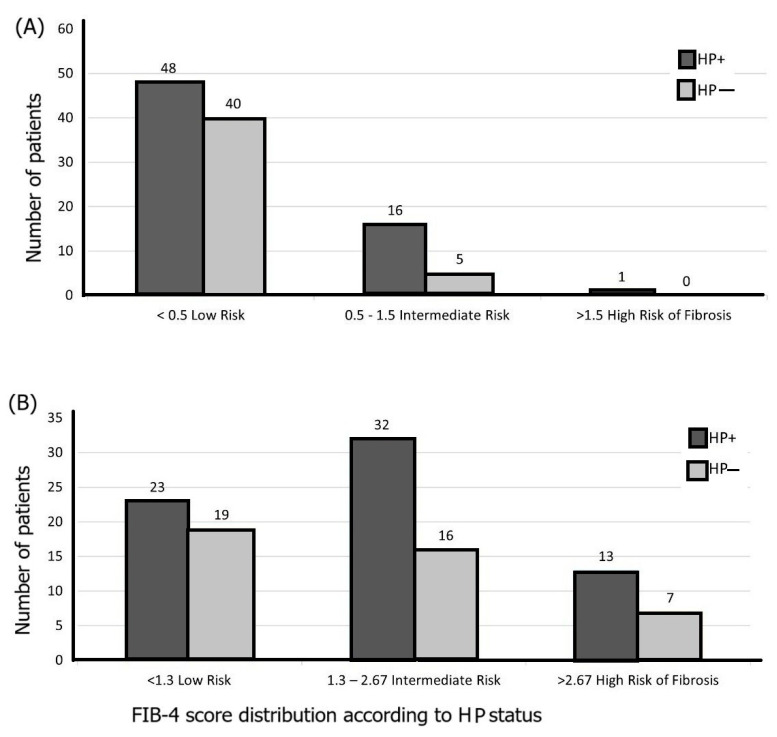

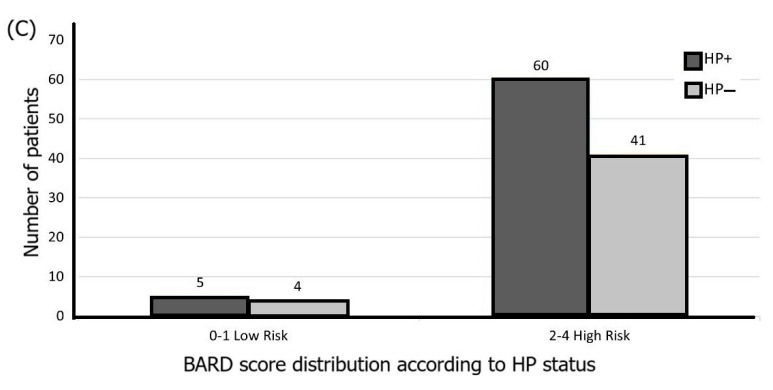
(**A**) APRI index, (**B**) FIB-4 index, and (**C**) BARD score distribution between patients according to *H. pylori* status.

Higher risk of liver fibrosis was noticed in patients with *H. pylori* infection versus patients without *H. pylori*, as shown in Figure 2A–C. Figure 2A,B highlights an intermediate risk for patients with *H. pylori* in developing liver fibrosis, when using the FIB-4 and APRI indexes, while when using the BARD score (Figure 2C), a higher risk of liver fibrosis was noticed in patients with *H. pylori*. Overall, *H. pylori* infection was associated with a higher risk of liver fibrosis, regardless of the score.

**Figure 3:**
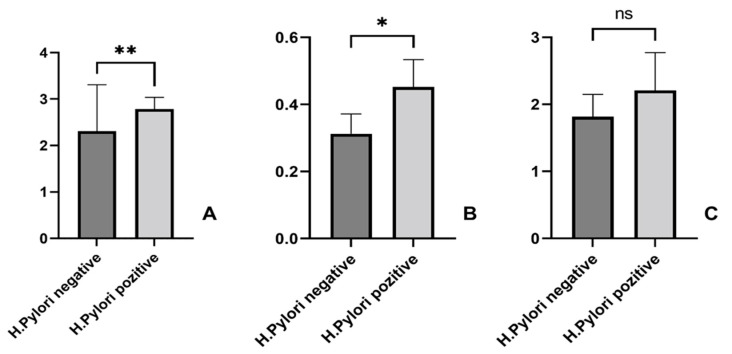
Correlation between liver fibrosis scores: the BARD score (**A**), the APRI index (**B**), and the FIB-4 index (**C**), and *H. pylori* status (** *p* = 0.005; * *p* = 0.03; ns = insignificant, *p* = 0.624).

As presented in Figure 3, we noticed significantly higher values of the BARD scores and the APRI indexes in patients with *H. pylori* and NAFLD, compared to *H. pylori*-negative patients. Among the 110 patients, 97 (88.1%) had BARD ≥ 2. We then performed an odds ratio analysis using the full sample size (n = 110). Among *H. pylori*-positive patients, approximately 57/65 (87.7%) had BARD ≥ 2, while among negative patients, 40/45 (88.9%) did. The resulting odds ratio was 0.89 (95% CI 0.27–2.92), indicating no significant difference between the two groups.

Moreover, statistically significant differences were noticed for BARD (** *p* = 0.005) and APRI index (* *p* = 0.03) when comparing *H. pylori*-positive and negative cases. Regarding the FIB-4 index, a statistically insignificant increase was observed in *H. pylori*-positive versus negative patients (*p* = 0.624).

### 3.3. H. pylori Patients’ Features and Liver Fibrosis Scores

Multiple linear regression analysis with BARD score as a dependent variable revealed a statistically significant regression coefficient parameter for age: β1 = 0.0155, *p* = 0.038, CI: (0, 0.03) and for *H. pylori* status: β3 = −0.456, *p* = 0.019, CI: (−0.839, −0.07). Gender and environment did not reach formal statistical significance. These results, as well as the parameter standard errors and 95% confidence intervals, are presented in Table 2.

Multiple linear regression involving the APRI score as a dependent variable also revealed statistically significant regression parameters for age: β1 = 0.004, *p* = 0.036, CI: (0, 0.008) and *H. pylori* status: β3 = −0.146, *p* = 0.0084, CI: (−0.254, 0.038). These parameters, as well as standard errors and confidence intervals, are presented in Table 3.

### 3.4. Associations Between Histopathological Features of the Gastric Lesions and Different Liver Fibrosis Scores

**Figure 4:**
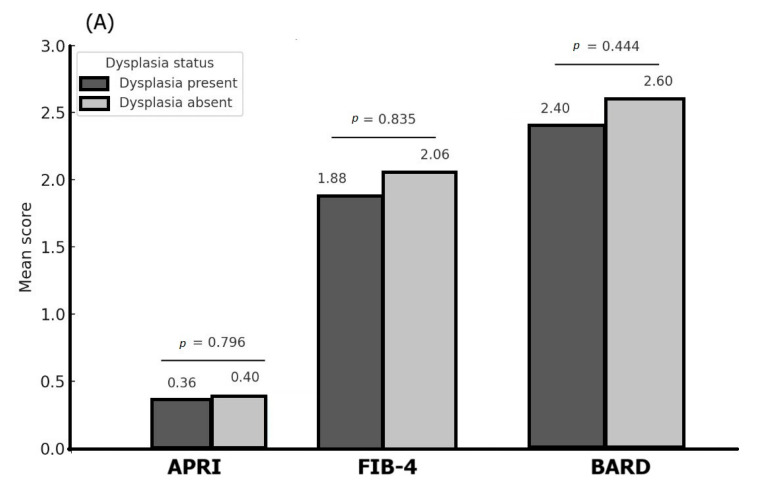

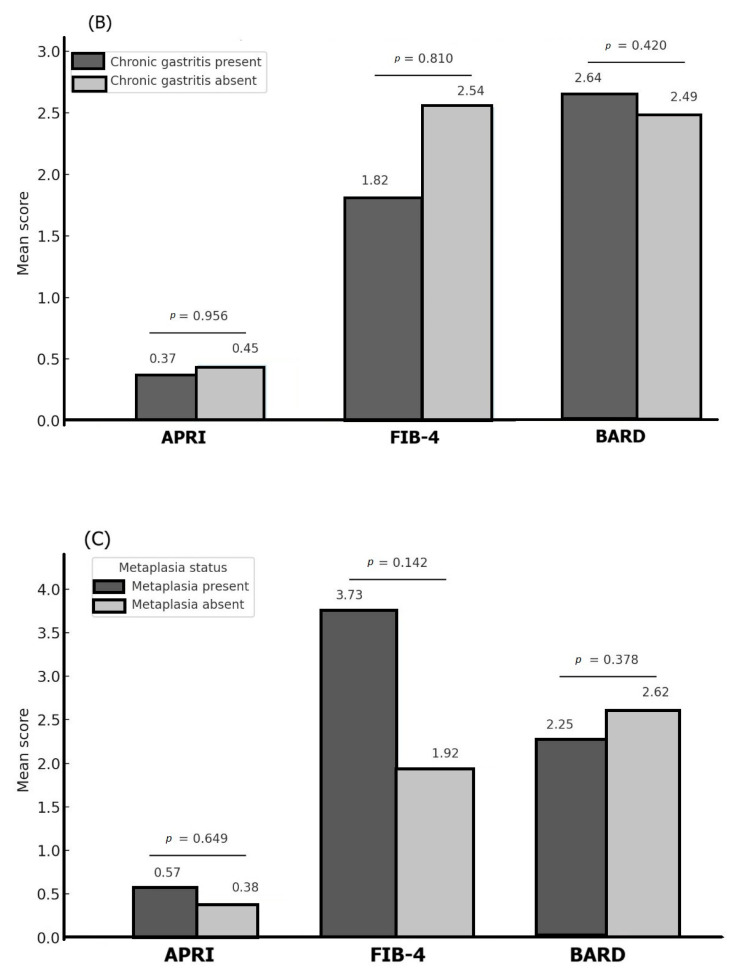
Association between different liver fibrosis scores and endoscopic features: (**A**) dysplasia; (**B**) chronic gastritis, and (**C**) metaplasia.

Figure 4A–C shows the association between different liver fibrosis scores (APRI, FIB-4, and BARD) and endoscopic features: dysplasia, chronic gastritis, and metaplasia.

Mean APRI scores were similar for patients with or without dysplasia (0.36 vs. 0.40, Mann–Whitney *p*-value = 0.796), while higher mean values of the FIB-4 index and the BARD score were noticed for patients without dysplasia, but with no statistical significance (*p* = 0.835, respectively, *p* = 0.444). On the other hand, APRI indexes were of similar value regardless the chronic gastritis status (*p* = 0.956), while mean FIB-4 indexes showed higher values (but not statistically significant) for patients without chronic gastritis (*p* = 0.810); mean BARD scores were higher for patients with chronic gastritis versus negative ones (*p* = 0.420). By contrast, the patients diagnosed with chronic gastritis presented higher BARD scores compared to negative results (*p* = 0.420). Similarly, the mean APRI, FIB-4, and BARD scores did not show statistically significant differences regarding metaplasia status, but a relatively large difference was noticed for the FIB-4 score (3.73 vs. 1.92 for patients with, respectively, without metaplasia, Mann–Whitney *p*-value = 0.142).

**Figure 5:**
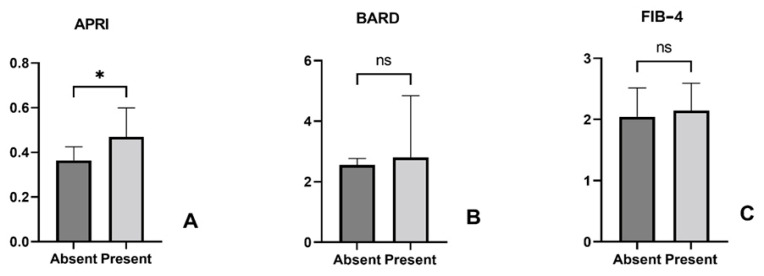
Association between liver fibrosis scores (APRI index (**A**), BARD score (**B**), and FIB-4 index (**C**)) and atrophic gastritis. (*—statistically significant, ns—statistically non-significant).

A different behavior was observed when evaluating the association between liver fibrosis scores and atrophic gastritis (Figure 5).

Patients diagnosed with atrophic gastritis of the gastric body exhibited significant higher APRI indexes (0.37 vs. 0.296, *p* = 0.038), while no significant association was identified with BARD score (3.0 vs. 2.6, *p* = 0.332) or FIB-4 index (2.01 vs. 1.39, *p* = 0.06) (Figure 4). Moreover, atrophic gastritis in the region of the antrum was not associated with any of the BARD score (*p* = 0.834), APRI (*p* = 0.337), and FIB-4 (*p* = 0.210) indexes.

## 4. Discussion

Our study highlights the possible role of *H. pylori* infection in liver fibrosis progression. To the best of our knowledge, this is the first study in Romania concerning the association between *H. pylori* infection and liver fibrosis scores. We demonstrated that patients infected with *H. pylori* exhibit higher liver fibrosis scores compared to negative. Moreover, *H. pylori*-positive patients diagnosed with NAFLD and hepatic fibrosis presented more severe gastric pathology compared to negative.

Based on multiple linear regression analysis, we highlighted that both BARD score and APRI index revealed a statistically significant association with age and *H. pylori* status. High prevalence rates of *H. pylori* infections were noticed all around the world, being influenced by a number of variables, including sex, age, socioeconomic status, geographical location, nutrition, and quality of life [30]. The global trend consists of a decreasing percentage in infected people during the last decade from 52.6% (95% CI, 49.6–55.6%) before 1990 to 43.9% (95% CI, 42.3–45.5%) in adults during 2015 through 2022 [31]. Although the prevalence results in our study (59,8% of the patients, mostly men) were different than previous studies, this may be due to the fact that the enrolled patients already suffered from dyspeptic symptoms, so that they were more likely to be diagnosed with the infection.

On the other hand, our patients were diagnosed with NAFLD, which might be associated with *H. pylori* [32]. An *H. pylori* infection may prevent leptin from being released by white adipose tissue, which in turn encourages hepatic stearoyl-CoA desaturase, speeding up the production of VLDL-C and fatty deposits in the liver tissue. It may also stimulate systemic inflammation and cause dyslipidemia [33]. In previous studies, *H. pylori* infection was associated with high total cholesterol and triglyceride values [34,35]. Previous studies devoted to the link between NAFLD and the presence of other infections suggested that certain infections with a severe evolution are more likely to occur in people with NAFLD [36]. Through immunological dysregulation and an elevated inflammatory milieu, it impairs the host’s resistance to pathogenic microorganisms. This increases the risk of severe infections, highlighting the importance of early NAFLD discovery and treatment [36]. A research study involving 202 patients with COVID revealed that those suffering from NAFLD experienced a prolonged duration of viral shedding and an increased likelihood of progressing to severe COVID-19 compared to those without the condition [37]. Additional research demonstrated the link between bacterial pneumonia or urinary tract infections and NAFLD. According to a retrospective analysis, the mortality rate was higher for NAFLD patients with community-acquired pneumonia [38].

In our study, the calculated liver fibrosis scores—BARD, APRI, and FIB4—were higher in *H. pylori*-positive patients. These features are in line with a study conducted on 584 morbid obese patients who underwent a gastric bypass. The percentage of *H. pylori*-positive patients had much higher APRI, NAFLD fibrosis score, and BARD scores compared to negative patients [39]. There are a number of possible explanations for the pathophysiology of the potential link between HP infection and NAFLD, including the creation of a pro-inflammatory state that causes the body to adopt a more lipogenic profile and a hormonal change that encourages the development of fibrosis and insulin resistance [40]. Our study showed that in patients with *H. pylori* and hepatic steatosis, the APRI score was the most statistically reliable in predicting the development of hepatic fibrosis and atrophic gastritis at the gastric body level. Although endoscopic features such as dysplasia, chronic gastritis, and metaplasia were not statistically associated with the BARD score, APRI or FIB-4 indexes, the patients diagnosed with atrophic gastritis of the gastric body exhibited significant higher APRI indexes.

Our study has a few limitations. First, the study was conducted in a single hospital center and with a relatively low number of participants. Finding patients who fit all the inclusion requirements was difficult. Furthermore, only symptomatic patients referred for a gastroscopy were enrolled, rather than patients undergoing regular examinations, which may be regarded as biased patient selection. Another limitation was in terms of the *H. pylori* diagnostic method, the RUT test being used to diagnose each patient. The specificities of other diagnostic techniques, such as stool antigen and serology, might be taken into account in a more precise manner. Another issue that could represent a limitation is the selection process, because only dyspeptic patients with NAFLD who underwent endoscopy with biopsies were taken into account. This selection criterion can be considered bias by restricting the study population.

Of course, this study leaves room for many other issues to be discussed. Taking into consideration the fact that atrophic gastritis was linked to hepatic fibrosis, one could raise the question whether hepatic fibrosis progresses along with the gastric lesions, knowing that atrophic gastritis is considered a later stage of *H. pylori* infection. The fact that gastritis of the gastric body was associated with liver fibrosis can also be taken into account, as the first stages of *H. pylori* colonization appear in the gastric antrum and only later in the body of the stomach [41]. Finally, further study would investigate whether hepatic fibrosis is a risk factor for *H. pylori* patients in developing precancerous gastric lesions such as metaplasia.

## 5. Conclusions

No statistically significant correlations were found between liver fibrosis scores (APRI index, BARD score, and FIB-4 index) and the presence of chronic gastritis, gastric metaplasia, and dysplasia. In contrast, the patients with atrophic gastritis in the gastric body presented a statistically significant higher APRI score of hepatic fibrosis. *H. pylori*-positive patients diagnosed with NAFLD and hepatic fibrosis presented more severe gastric pathology compared to negative ones: significantly higher values of the BARD score and APRI index were observed in *H. pylori*-positive and NAFLD patients versus negative ones. Although the study was conducted on a small scale, our work offers an intriguing view into unexplored areas in the field of *H. pylori* particularities. By exploring the complex realm of infection and the complexities of histopathology, we demonstrated a significant association between *H. pylori* and high values of hepatic fibrosis scores in patients with NAFLD suffering from gastritis. Deciphering the progression of gastric biopsies and the changes in hepatic fibrosis scores before and after eradication is crucial for the future.

## Figures and Tables

**Table 1:** Patients’ baseline characteristics and *H. pylori* status (M—mean value, SD—standard deviation).

Variable	Overall (M ± SD)	*H. pylori*+ (M ± SD)	*H. pylori* − (M ± SD)	*p*-Value (*HP−* vs. *HP*+)
Age (years)	62.3 ± 12.7	61.9 ± 12.0	63.0 ± 13.8	0.663
BMI (kg/m^2^)	28.8 ± 8.2	29.9 ± 7.8	25.9 ± 8.4	0.058
ALT (U/L)	28.4 ± 21.2	32.1 ± 21.4	23.0 ± 20.0	0.025
AST (U/L)	33.7 ± 21.1	38.1 ± 23.4	27.5 ± 15.6	0.005
Platelet count (10^3^/µL)	244.9 ± 92.5	248.8 ± 103.9	239.3 ± 73.8	0.575
Fasting glucose (mg/dL)	110.4 ± 44.0	110.8 ± 34.6	110.0 ± 55.1	0.928
Total cholesterol (mg/dL)	181.1 ± 50.1	188.9 ± 52.7	169.0 ± 43.8	0.038
Triglycerides (mg/dL)	139.5 ± 83.4	150.8 ± 98.4	122.3 ± 49.4	0.052
HDL cholesterol (mg/dL)	44.4 ± 20.4	43.7 ± 23.7	45.9 ± 10.9	0.545
LDL cholesterol (mg/dL)	107.1 ± 35.6	108.6 ± 33.6	104.4 ± 39.5	0.606
Diabetes mellitus (n, %)	41 (45.1%)	27 (42.2%)	14 (51.9%)	0.538

**Table 2:** Multiple linear regression table for BARD score. β0–β4 represent the regression coefficients for each variable. The estimate for each coefficient, along with the standard error, 95% confidence interval, and *p*-value, is shown. Female gender, *H. pylori*-negative, and urban environment are the default variables.

Parameter Estimates	Variable	Estimate	Standard Error	95% CI (Asymptotic)	*p*-Value
β0	Intercept	1.874	0.487	0.907–2.841	0.0002
β1	Age	0.0155	0.007	0–0.03	0.0389
β2	Gender (female = 0)	0.2794	0.188	−0.09–0.653	0.141
β3	*H. pylori* (negative = 0)	−0.4561	0.191	−0.839–−0.0	0.019
β4	Environment (urban = 0)	−0.330	0.191	−0.710–0.05	0.088

**Table 3:** Multiple linear regression table for APRI score. β0–β4 represent the regression coefficients for each variable. The estimate for each coefficient, along with the standard error, 95% confidence interval, and *p*-value, is shown. Female gender, *H. pylori*-negative, and urban environment are the default variables.

Parameter Estimates	Variable	Estimate	Standard Error	95% CI (Asymptotic)	*p*-Value
β0	Intercept	0.189	0.138	−0.085–0.464	0.175
β1	Age	0.004	0.002	0–0.008	0.036
β2	Gender (female = 0)	−0.047	0.053	−0.153–0.058	0.378
β3	*H. pylori* (negative = 0)	−0.146	0.054	−0.254–−0.038	0.0084
β4	Environment (urban= 0)	0.014	0.054	−0.094–0.122	0.797

## Data Availability

All data are available in the County Emergency Clinical Hospital of Oradea—Bihor, Romania, including databases and consultation registers from the Gastroenterology Department, and the paraffin-embedded tissue blocks from the Pathology Department.
